# Urbanization leads to asynchronous homogenization of soil microbial communities across biomes

**DOI:** 10.1016/j.ese.2025.100547

**Published:** 2025-03-17

**Authors:** Bangxiao Zheng, Nan Hui, Ari Jumpponen, Changyi Lu, Richard Pouyat, Katalin Szlavecz, David A. Wardle, Ian Yesilonis, Heikki Setälä, D. Johan Kotze

**Affiliations:** a School of Agriculture and Biology, Shanghai Jiao Tong University, Shanghai, 200240, PR China; b Faculty of Biological and Environmental Sciences, Ecosystems and Environment Research Programme, Niemenkatu 73, FI-15140, Lahti, University of Helsinki, Finland; c Center for Ecology & Health Innovative Research, Xiamen University of Technology, Xiamen, 361024, PR China; d 433 Ackert Hall, Division of Biology, Kansas State University, Manhattan, KS66506, USA; e Key Laboratory of Urban Environment and Health, Ningbo Urban Environment Observation and Research Station, Institute of Urban Environment, Chinese Academy of Sciences, Xiamen, 361021, PR China; f Zhejiang Key Laboratory of Urban Environmental Processes and Pollution Control, CAS Haixi Industrial Technology Innovation Center in Beilun, Ningbo, 315830, PR China; g Emeritus USDA Forest Service, NRS, Affiliate Faculty Department of Plant and Soil Sciences, University of Delaware, Newark, DE, 19716, USA; h Department of Earth and Planetary Sciences, Johns Hopkins University, 3400 N. Charles St, Baltimore, MD, 21218, USA; i Department of Ecology and Environmental Sciences, Umeå University, Umeå, Sweden; j USDA Forest Service, Baltimore Field Station, Maryland, USA; k Shanghai Urban Forest Ecosystem Research Station, National Forestry and Grassland Administration, Shanghai 200240, China; l Shanghai Yangtze River Delta Eco-Environmental Change and Management Observation and Research Station, Ministry of Science and Technology, Ministry of Education, Shanghai 200240, China

**Keywords:** Asynchronous homogenization, Bacterial and fungal community, Disturbance gradient, Taxon and trait composition, Urbanization

## Abstract

Soil bacterial and fungal communities play fundamental roles in biogeochemical cycles and ecosystem stability. Urbanization alters soil properties and microbial habitats, driving shifts in community composition, yet the divergent responses of bacteria and fungi and their ecological consequences remain inadequately understood. To elucidate these differential responses, we investigated soil bacterial and fungal communities along an urbanization gradient, ranging from undisturbed reference forests to urban parks, across three distinct climatic regions. To capture different disturbance intensities, urban parks were classified by tree age into old parks (>60-year-old trees) and young parks (10–20-year-old trees). Climate had a strong influence on soil microbiota, yet urbanization still significantly altered both bacterial and fungal communities in all regions. Urban disturbances homogenized soil microbial communities: average similarity among bacterial communities increased from ∼79 % in forests to ∼85 % in young urban parks, indicating substantial homogenization, whereas fungal communities showed little homogenization. Urbanization also homogenized microbial functional traits, with a greater reduction in trait dissimilarity for bacteria than for fungi. Bacterial communities exhibited high adjustability to urban conditions, dominated by generalist taxa (∼90 %), whereas fungal communities consisted mostly of specialists (∼83 %). Despite these asynchronous responses—bacteria adjusting and homogenizing more than fungi—overlapping functional traits between bacteria and fungi help maintain functional resilience in urban ecosystems.

## Highlights

• Urbanization increases microbial community similarity from ∼79 % to ∼85 % across biomes.
• Bacteria homogenize more than fungi, with functional traits converging faster in bacteria.
• Urban soils host ∼90 % bacterial generalists but ∼83 % fungal specialists, revealing adaptive contrasts.
• Despite divergent responses, shared microbial traits sustain ecosystem resilience.

## Graphical abstract

**Figure:**
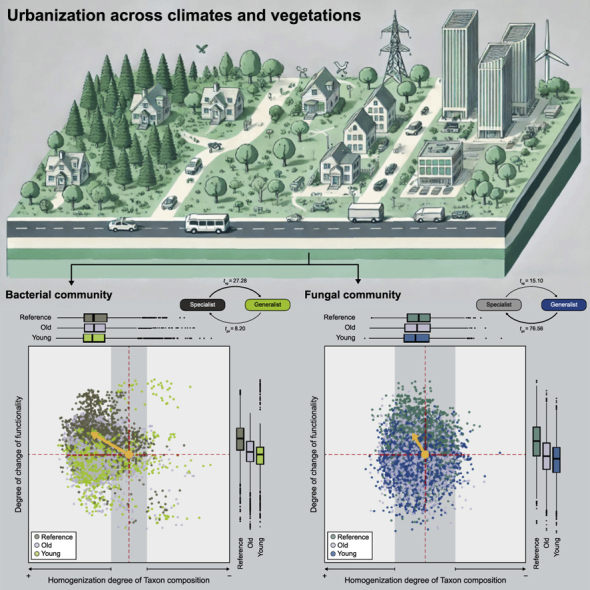

## 1 Introduction

Urban land use typically disturbs soil structure, increases soil nutrient concentrations, elevates heavy metal levels, and degrades and fragments habitats, thereby favoring species adapted to these altered conditions [1]. Additionally, urbanization often homogenizes species assemblages [2], resulting in increasingly similar urban communities of plants, animals [3], and microbes [[4], [5], [6]]—which, in turn, alters ecosystem functioning [7]. The homogenization of ecosystem functions has been reported to correlate with changes in plant and animal communities [[8], [9], [10], [11]]. However, studies often overlook bacterial and fungal communities in urban habitats, despite their critical roles in ecosystem processes and resilience to disturbance [12]. Soil bacteria and fungi share perform key functions in nutrient mineralization and organic matter (OM) decomposition [13,14], though their roles often differ at various stages of substrate decomposition [15,16]. Bacterial and fungal responses to disturbances likely diverge because of the greater adaptive potential of bacteria—driven by, for example, shorter generation times and more frequent horizontal gene transfer (HGT)—compared to fungi [17,18]. Such differences may result in asynchrony between disturbance-induced community dynamics and the functional potentials of these taxa—similar to macro-organisms, whose species respond variably to urban disturbances due to their distinct life-history traits [[19], [20], [21]].

Community homogenization due to urbanization arises from both press (continuous stressors) and pulse (short-term disturbances) events, driven by the restructuring of native taxa and the establishment of invasive and exotic generalists, which can lead to the extirpation of endemic specialists [5,22]. However, only a few studies have investigated whether such compositional changes may also homogenize bacterial and fungal functional potentials. To explore the sensitivity of soil microbes and their functional attributes to an urban disturbance gradient, this study tested the following hypotheses: Across climatic regions, urban press and pulse disturbances—along with their concomitant system alterations, including soil structure simplification—(1) homogenize bacterial and fungal communities along urban disturbance gradients across biomes, (2) differentially homogenize bacterial and fungal communities, and (3) drive divergent functional trait compositions because of distinct bacterial and fungal adaptive strategies to soil disturbances [23]. Additionally, (4) community and functional trait homogenization would be asynchronous, as pulse and press disturbances and the resultant compositional changes do not necessarily translate into changes in functional potentials due to redundancies in diverse microbial communities [10,24]. Addressing these questions will enhance our understanding of whether ecosystem functions would be retained or some would be lost due to global urbanization.

We investigated the potential homogenization of the soil microbiota (here bacterial and fungal communities and their functions) along an urban disturbance gradient spanning reference forests (natural stands with minimal anthropogenic disturbance) to old (intermediate disturbance) and young (heavily disturbed due to recent park establishment) urban parks. To assess whether the strong influence of geographic region on biota [25] affects the degree of compositional and functional homogenization, we studied three cities across three climatic biomes: boreal (Lahti, Finland), temperate (Baltimore, USA), and tropical (Singapore). Across and within biomes, plant communities differ in the quality of litter they produce [26,27]. Given that bacteria and fungi play different roles in litter decomposition, we sampled soils beneath different vegetation types at each study site: urban parks, with trees producing labile or recalcitrant litter and lawns producing labile litter, and reference forests, with trees producing labile or recalcitrant litter. We previously observed that vegetation type influences plant–soil interactions [28,29] and that urbanization shapes the composition and functional traits of microbial communities [30], leading to the expectation that microbial communities and their functional abilities would shift accordingly. In this study, we (i) used Illumina MiSeq-sequenced bacterial and fungal metabarcodes to characterize their community composition and (ii) applied GeoChip 5.0 gene arrays to analyze microbial functional traits, including those related to carbon and nutrient cycling [31]. Our study was explicitly driven by whether the shifts in and the homogenization of community composition would be mirrored by those of functional potentials and gene abundances [32,33]. We analyzed the asynchrony between community and functional trait composition using multivariate dispersion (PERMDISP) to address this research question. Additionally, we analyzed changes in community composition and functional potentials within the soil microbiota from an evolutionary perspective to evaluate the degree of species turnover under disturbance.

## 2 Materials and methods

### 2.1 Experimental design

The detailed experimental design is described in Kotze's paper [29]. Briefly, we sampled soils in three cities across three climatic zones: boreal (Lahti, Finland), temperate (Baltimore, USA), and tropical (Singapore). While we refer to these as boreal, temperate, and tropical zones or cities throughout the paper, we acknowledge that the selected cities may not fully represent their respective climatic zones. In each biome, managed urban parks representing two age groups were selected: young parks (trees aged 10–20 years, *n* = 5) and old parks (trees aged more than 60 years, up to 200 years, *n* = 5). A natural to semi-natural forest (∼100 years old, *n* = 5) was chosen as a reference. In parks, three plant types were categorized based on their litter decomposability [26]: trees producing recalcitrant litter, trees producing labile litter, and lawns with highly labile grasses and forbs. In reference forests, the lawn was absent, but the same recalcitrant and labile tree species as those in parks were selected. A total of 120 plots were established across three climatic zones, including 5 replicates for each of the 3 plant types in each old and young park and 5 replicates of recalcitrant and labile tree species in reference forests (https://www.google.com/maps/d/edit?mid=1XLYE2s4PTbWzW8lAsqdqxxH6cb9UUzV&ll=21.28548949556766 %2C13.624695500000037&z=3). Urbanization in this study is quantitatively defined not only by immediate physical disturbances but also by the duration of such impacts, as reflected in the age of urban parks. Older parks represent a longer history of urban influence on plant and soil ecosystems, whereas reference forests are minimally impacted by urbanization. To assess urbanization, we used park age as a proxy for the duration and intensity of urban impacts on ecosystems. This approach was complemented by comparisons to reference forests, which serve as control sites with negligible urban influences (see also [29]). To focus on the effects of urbanization, microbial variables from different vegetation types within each park were averaged, summarizing the influence of vegetation type on soil conditions and microbial communities across climatic regions [29]. Soil sampling followed a standardized protocol described previously [28,34,35]. Fresh soils were sieved to remove rocks and debris, preserved in 20 mL LifeGuard Soil Preservation Solution (QIAGEN, Germany), and stored at −70 °C prior to deoxyribonucleic acid (DNA) extraction.

### 2.2 Sequencing and GeoChip measurements

The detailed methodology is described in a previous study [30]. Briefly, DNA was extracted using the DNeasy PowerMax Soil Kit (QIAGEN, Hilden, Germany), quantified using the Qubit RNA IQ Assay Kit (Invitrogen, Massachusetts, USA), and quality verified using 1 % agarose gel electrophoresis. The DNA samples were then diluted to 1.5 ng μL^−1^ for amplicon sequencing. The bacterial 16S rRNA V4–V5 (515F/907R) and fungal ITS1 (ITS1F/ITS2) regions were amplified by adding barcodes and adaptor sequences and then sent to the Novogene UK Sequencing Center for library construction and sequencing on the Illumina MiSeq 2500 platform. A total of 22.02 million 16S rRNA and 22.99 million internal transcribed spacer (ITS) metabarcoding amplicons were received and transformed to operational taxonomic units (OTUs) through demultiplexing (QIIME2-2020.2) [36] and denoising (DADA2) [37]. The OTUs were then annotated using Greengenes [38] and UNITE [39] databases. Sequences are archived in the NCBI Sequence Read Archive (SRA) under the project PRJNA633748.

The purified DNA samples were further analyzed for functional gene intensity measurements using GeoChip 5.0 (Glomics Inc.) [40]. A positive spot indicates an effective probe detection for a certain functional gene of a taxon (species, subspecies, or strain) and was received after labeling, hybridization, imaging, and denoising (http://ieg.ou.edu/microarray), as previously described [41,42]. We focused on functional traits related to carbon (C), nitrogen (N), phosphorus (P), and sulfur (S) cycles. The corresponding datasets are accessible in the NCBI Gene Expression Omnibus (GEO) under the project PRJNA722405. Among trait-based approaches, the community-weighted mean (CWM) method was selected due to its lower statistical error and higher stability [[43], [44], [45]]. CWM analysis integrates GeoChip gene intensities with sequence taxon abundances as functional traits, based on the following assumptions: (1) the spot intensity for each trait represents gene abundance per gene per taxon [46]; (2) the major taxon associated with a given function can be reliably detected using both amplicon sequencing and GeoChip arrays [47]; and (3) the amplicon sequencing results of 16S and ITS can be used as a credible measure of the relative abundances of taxa, despite known reproducibility challenges [48]. The CWM trait *j* per plot *k* can be calculated as

(1) $$\begin{array}{c} {CWM}_{j,k}=\sum \varphi_{i,k}\lambda_{i,j,k} \end{array}$$

where *φ*_*i,k*_ is the abundance of taxon *i* in plot *k* (derived from amplicon sequencing) and *λ*_*i,j,k*_ is the average trait abundance *j* of taxon *i* in plot *k* (derived from GeoChip analysis). GeoChip-derived functional genes serve as indirect markers of microbial functional traits, and the calculated trait abundance should be interpreted as indicators of potential physiological or biochemical functions. However, determining a direct causal relationship between such traits and ecosystem processes requires further investigation [49,50].

By analyzing the CWM traits, we examine functional redundancy within soil microbial communities. Functional redundancy—the ability of different microbial species to perform the same ecological functions—is crucial for maintaining ecosystem resilience in urbanized environments. This redundancy ensures the continuity of ecological processes despite species turnover, contributing to functional stability under urban pressures.

### 2.3 Statistical analyses

The statistical analyses were performed using R version 3.6.3 [51]. Graphics were generated using R (ggord [52] and ggplot2 [53]) and Illustrator 2019 (Adobe). The normality of all variables, including edaphic properties, taxa, and trait abundances, was verified via the Kolmogorov–Smirnov and Shapiro–Wilk tests, and when necessary, data were normalized using Hellinger or *z*-score transformations (*decostand*, vegan) [54]. Between-group differences between species and trait abundances were analyzed using permutational multivariate analysis of variance (PERMANOVA, *adonis*, vegan). The significant dispersion of microbial communities was analyzed using homogeneity of multivariate dispersions (PERMDISP, *betadisper*, vegan); the resulting dispersions were illustrated in principal co-ordinates analysis (PCoA) space with 95 % confidential ellipse (*betadisper*, vegan). Tukey's honest significant difference (HSD) method was used to evaluate the significance of between-group differences (*TukeyHSD.betadisper*, vegan). The random forest algorithm (randomForest) [55,56], a decision tree-based classification method [57], was employed to analyze the influence of urbanization gradients (ranked by contribution) on taxa and functional traits. The top 35 contributors (ASVs or traits) were clustered (pheatmap) [58] to elucidate whether the effect of urbanization remained dominant despite strong climatic biome impacts [59].

***Structural equation models (SEMs)*.** SEMs were constructed to examine the relationships among soil properties, bacterial and fungal community diversity, and functional traits (see Hypothesis 3). Edaphic properties, including pH, bulk density (BD), organic matter (OM), C content, N content, C/N ratio [29], and plant root density (unpublished), were used to in this analysis. Variables such as longitude, edaphic properties, root density, bacterial and fungal diversity indices (i.e., richness, Shannon-diversities, evenness), and bacterial and functional traits were used to test the direct and indirect relationships. Model fit was tested using the χ^2^ test and root mean square error of approximation (RMSEA) index, with the following thresholds: χ^2^/*df* < 3, RMSEA <0.08, and *p* value of close fit (PCLOSE) > 0.05 [60]. Different combinations of these variables were tested, with non-significant relationships iteratively removed or substituted until stable SEMs were achieved. Note that the constructed SEMs in this study rely solely on the observed variables, which provide insights into microbial community composition and function. However, these models cannot be used to test mechanisms and causations, which need to be addressed through laboratory experiments.

***Ecological process estimation*.** To phylogenetically evaluate whether taxon and trait compositions were homogenized due to urbanization, a standard framework for quantifying ecological processes was employed [61]. These processes included “determinism” mechanisms (homogeneous and heterogeneous selection by abiotic environments) and “stochasticity” mechanisms (i.e., historical contingencies) that are driven by, for example, chance colonization and random extinction, including homogenizing dispersal, dispersal limitation, and drift [62]. A fundamental assumption in this framework is that phylogenetic distances among taxa reflect discrepancies in their ecological niches (phylogenetic signal) [61,63]. To test this assumption, we performed a Mantel correlogram (*mantel.correlog*, vegan) analysis, comparing the matrices of each edaphic property (pH, bulk density, organic matter, C, N, and C/N ratio). The results confirmed that soil properties influenced phylogenetic distances, which fulfills the precondition (Supplementary Material Fig. S2). Phylogenetic distances were further used to calculate *β*NTI (*β*-nearest-taxon-index) [61], which measures deviation of *β*MNTD (*β*-mean-nearest taxon distance) [64] from a null model expectation. Additionally, we computed RC_bray_, the abundance-based (Raup-Crick) *β*-diversity, based on pairwise Bray-Curtis dissimilarity [65]. Rare species (defined as species that occupy fewer than 10 % of all study plots) [66,67] were removed to avoid qualitative error rates and technical noise [68,69], leaving 5903 bacterial and 983 fungal OTUs for the analysis. Ecological processes were categorized using the following criteria: if the observed *β*MNTD deviation is greater (*β*NTI > 2) or less (*β*NTI < −2) than the null expectation, the assemblage will be considered heterogeneous or homogeneous selection, respectively. The *β*MNTD deviations that satisfy the null hypothesis (−2 < *β*NTI < 2) will be further classified into RC_bray_ (dispersal limitation with RC_bray_ > 0.95), drift (if RC_bray_ falls between −0.95 and 0.95), and homogenizing dispersal (when RC_bray_ is less than −0.95) [61,70]. To analyze the independence of phylogenetic and functional homogenization, *β*NTI was used as a proxy for phylogenetic turnover, while trait turnover was calculated using the functional dissimilarity index [71,72]:

(2) $$\begin{array}{c} \beta_{\text{diss}}=\frac{\sum \left|\lambda_{i,j,m}-\lambda_{i,j,n}\right|}{\sum \left(\lambda_{i,j,m}+\lambda_{i,j,n}\right)} \end{array}$$

where *β*_diss_ is functional dissimilarity, and *λ*_*i,j,m*_ and *λ*_*i,j,n*_ are the average trait abundance *j* of taxon *i* at two sites (*m*, *n*).

***Evolutionary analysis*.** To elucidate the evolutionary mechanism underlying microbial community homogenization, we classified taxa into generalists (species capable of surviving in diverse habitats) and specialists (species adapted to specific environments) [73]. Niche width (or niche breadth) [74] was calculated as

(3) $$\begin{array}{c} B_{j}=\frac{1}{\sum_{i=1}^{N}P_{i,j}^{2}} \end{array}$$

where *B*_*j*_ indicates the niche width and *P*_*i,j*_ is the proportion of species *j* in plot *i* (Supplementary Material Fig. S3a). Generally, taxa/species with higher *B*-values are considered habitat generalists, while those with lower *B-*values are considered habitat specialists [75]; however, the cut-off criterion is usually arbitrary. Niche width refers to the range of resources used by communities, while the trait per species in communities suggests the potential of an individual to utilize resources. A positive relationship between niche width and trait per species indicates that species broaden their functional traits to adapt to diverse environments (i.e., generalists), while a negative relationship suggests that some functions of species are closely associated with a given environment (i.e., specialists). Based on this, a threshold niche width of 9 was chosen to distinguish generalists from specialists for both bacterial and fungal communities (Supplementary Material Fig. S3b). To determine the evolutionary characteristics of generalists and specialists, such as speciation, extinction, and state-transition rates, a binary-state model (BiSSE, binary-state speciation and extinction) [76] was used. For both bacterial and fungal phylogenetic trees, *make.bisse* (diversitree) [77] was run twice: once to search for the starting point of the simulation and again to calculate the maximum likelihood estimation of rates. Then, 1000-step Markov chain Monte Carlo (MCMC) simulations were performed (*mcmc*, diversitree) to assess the stability of the final estimation.

## 3 Results and discussion

### 3.1 Climatic and urbanization impacts

**Fig. 1:**
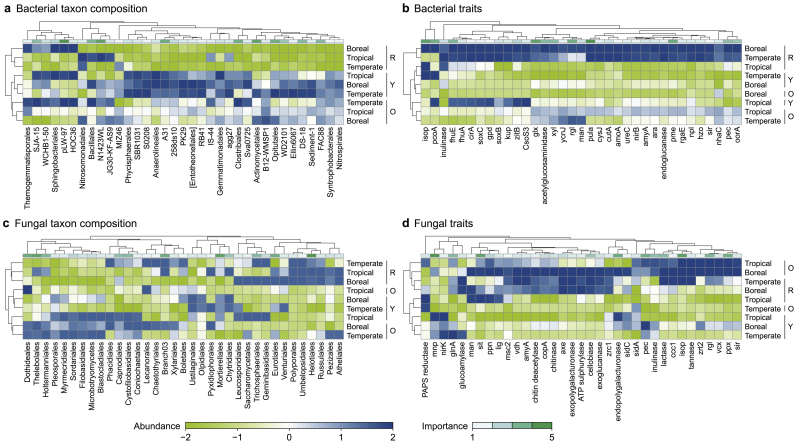
Urbanization (here defined as a disturbance gradient from reference forests to old and young parks) has a strong effect on clustering microbial communities (by random forest analyses). The most influencing species and genes for community and trait composition structuring that are compliant with disturbance gradients were ranked by random forest algorithm, and we selected the top 35 species and genes for clustering analyses. Heatmaps show the taxon (**a**, **c**) and trait (**b**, **d**) composition of bacterial (**a**–**b**) and fungal (**c**–**d**) communities. The “abundance” legend represents the abundance of that taxon or trait of the bacterial and fungal communities, increasing from light green to dark blue. The index of “importance” indicates the relationships between taxon or trait abundance and disturbance gradient, suggesting the contribution/importance of some taxa or traits in structuring composition along the disturbance gradient (reference forest [F], old parks [O], young parks [Y]). Taxon and trait abundances were transformed by z-score standardization. The clustering of rows is based on Bray-Curtis distances. The clustering of taxa or traits above each figure is also generated based on Bray-Curtis distances.

Climate is often a predominant factor controlling terrestrial communities [25]. As expected, in this study, both taxonomic and functional trait compositions of soil bacteria and fungi differed among climatic zones (biome effect, *p* < 0.001, Supplementary Material Table S1). Despite this strong climatic effect [59], urban disturbances significantly structured top-ranked microbial taxa (based on random-tree analysis) and functional traits along a gradient from reference forests to old parks to young parks (Fig. 1, Supplementary Material Table S1). This pattern suggests urban homogenization of microbial communities. In contrast, vegetation type (plants producing recalcitrant or labile litter) had minimal effect on microbial communities or functional potential (*R*^2^ = 0.012–0.021, *p* = 0.036–0.728), as indicated by the low within-group variation among the bacterial community, functional trait composition, and fungal traits. However, independent of the biome, fungal communities differed by vegetation type, albeit weakly (PERMANOVA, Supplementary Material Table S1).

**Fig. 2:**
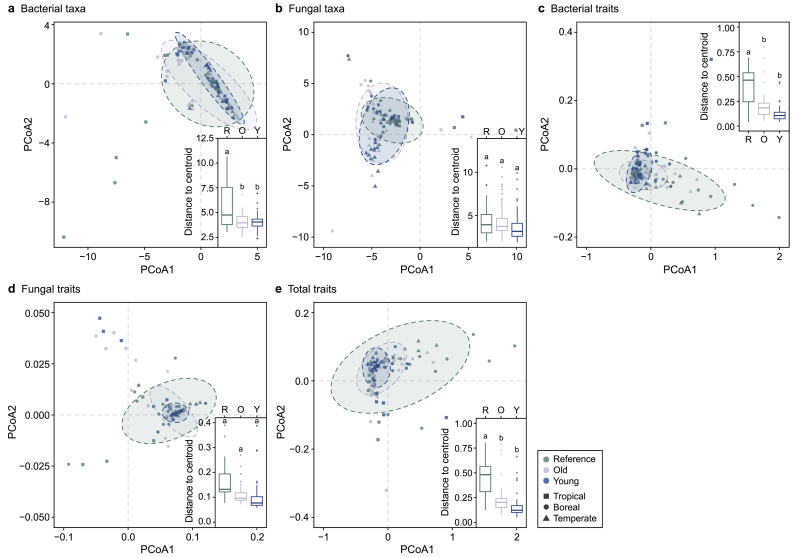
Dispersion analyses (PERMDISP) of bacterial taxon (**a**) and trait (**c**) composition, fungal taxon (**b**) and trait (**d**) composition, and total trait composition (**e**) along a disturbance gradient from reference forest (R, little human disturbance) to old parks (O, moderate disturbance) and young parks (Y, heavy disturbance) across biomes. The significant decrease in distance to the centroid estimated by PERMDISP from R to O and Y (e.g., panels **a**, **c**, and **d**) suggests taxon or trait composition homogenization with urban disturbances, which can also be observed from the decreased areas of the ellipses. Within-group variation is measured as the distance of plots to their group centroid (boxplots, solid lines indicate medians and quartiles). Different letters indicate significant levels at *p* < 0.05 by Tukey's HSD test. The ellipses indicate 95 % confidence intervals based on Bray-Curtis similarity in PCoA ordinations.

**Fig. 3:**
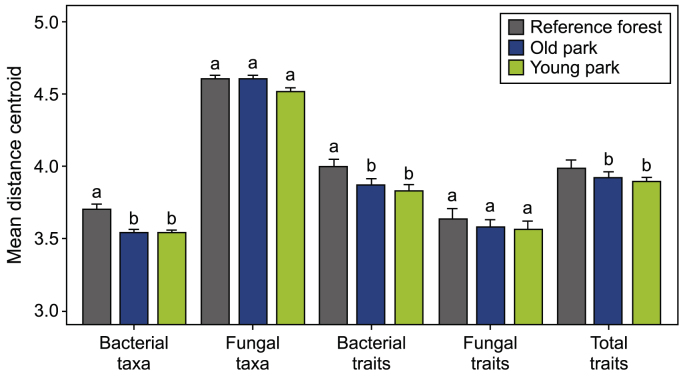
The mean distance to centroid estimated by dispersion analyses (PERMDISP) of bacterial taxon, fungal taxon, bacterial trait composition, fungal trait composition, and total trait composition along a disturbance gradient from reference forest (litter human disturbance) to old parks (moderate disturbance) and young parks (heavy disturbance) across biomes. The dispersion estimation is based on the Bray-Curtis distance and is presented in PCoA ordinations. The value of the mean distance to the centroid is a natural logarithm transformed. Letters indicate the within-group across all urban categories across latitudes difference at a significance level of *p* < 0.05 by one-way analysis of variance (ANOVA).

Bacterial and fungal communities responded differently to urban disturbances. Across biomes, bacterial communities and functional traits were more homogenized (i.e., less dispersed) in urban parks than in reference forests (closer to the centroid; small panels in Fig. 2a–c, Supplementary Material Table S2). However, homogenization of the bacterial community was asynchronous with that of its functional traits, as indicated greater dispersion in community composition than in traits (Fig. 3, Supplementary Material Table S2). In contrast, neither fungal communities nor their functional traits differed in dispersion, although the dispersion of trait composition decreased from reference forest to urban parks (Fig. 2, Fig. 3, Supplementary Material Table S2). Overall, the total trait composition was significantly homogenized by urban disturbances (Fig. 2, Fig. 3; PERMDISP, *F* = 18.296, *p* = 0.001, Supplementary Material Table S2), an effect driven primarily by the bacterial community. This finding suggests substantial functional redundancy in the soil microbiome of disturbed urban soils. Although fungi generally seem more sensitive to urban disturbances than bacteria [5,78], we argue that their greater genomic potential afforded by the large genomes, which maintain a greater composition of potential traits, may obscure detectable urban effects. As a result, we did not observe any clear impacts of urban disturbances on soil fungi. In summary, urban disturbances primarily drive bacterial community homogenization and functional homogenization, partly supporting Hypothesis 1 and indicating a convergence of bacterial communities and their functions in urban habitats across three climatic zones. This trend mirrors previously reported soil physicochemical properties in urban habitats across climatic zones [29].

The divergent bacterial and fungal communities responses to the urban disturbance gradient support Hypothesis 2 and highlight discrepancies between community and trait compositions, supporting Hypothesis 4. Urban disturbances impacted fungi and bacteria differently, with fungi exhibiting less trait homogenization than bacteria. These differing responses are attributable to shared traits that enhance the overall microbial system's resilience, rather than favoring one group over another [79]. We anticipated that urban homogenization would lead to less diverse microbial communities in simplified and disturbed urban soils, whereas resource-rich forests with greater spatial heterogeneity would have more diverse communities [29]. However, while there was compositional homogenization of the communities, there was asynchrony between community composition and functional traits, indicating that there are still many shared, redundant functions in urban and homogenized soils. Bacterial diversity was higher in old parks compared to the reference forests, whereas fungi showed the opposite trend. This suggests that bacteria benefit from urbanization by expanding their collective functional potential, a surprising finding given the simplified and disturbed soil conditions.

### 3.2 Bacterial and fungal community responses

**Fig. 4:**
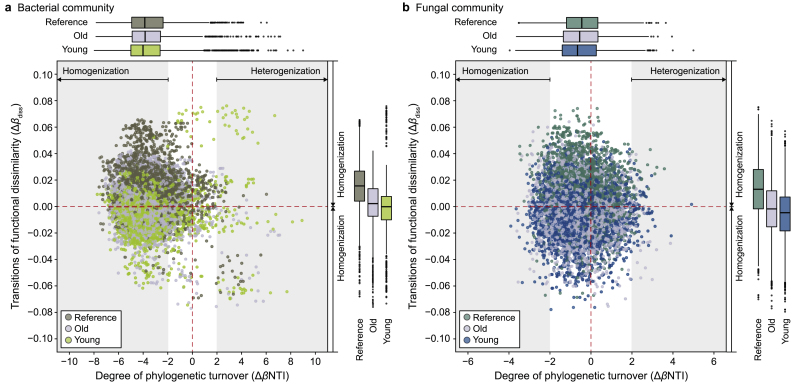
Comparisons between phylogenetic turnover and changes in functional dissimilarities of bacterial (**a**) and fungal (**b**) communities (*n* = 7140) with urbanization across biomes. Compositional transitions are indicated by the degree of phylogenetic turnover (Δ*β*NTI). Turnover below Δ*β*NTI < −2 or above Δ*β*NTI > 2 indicates compositional homogenization or heterogenization, respectively; turnover within the range −2 < Δ*β*NTI < 2 indicates the phylogenetic status of homogenization limitations (see Supplementary Material Table S3). Changes of functional dissimilarity (Δ*β*_diss_) along the vertical axis indicate the degree of variation of traits.

To further explore whether shifts in the microbial functional trait composition along an urban disturbance gradient relates to speciation or extinction, we analyzed functional compositional changes (Δ*β*_diss_) and phylogenetic turnover (Δ*β*NTI). The latter quantifies the evolutionary distances among species assemblages and is affected by geographic and environmental distance between sites [80]. This enables identifying which of the two —the arrival of new taxa or extinction—is the primary cause of species turnover between abiotic environments and stochastic dispersion across biomes. Bacterial trait composition homogenized with urbanization, as indicated by the decrease in trait dissimilarity from reference forests to old and young parks across biomes (Fig. 4a, *y*-axis). Based on Δ*β*NTI estimates, more than 79 % of the ASVs of the entire bacterial community were under homogeneous selection (Fig. 4a, *x*-axis; Supplementary Material Table S3), an ecological selection with little variation in community composition. Abiotic factors, including soil temperature, pH, and moisture, commonly lead to the homogeneous selection of the soil community [81,82]. Structural equation modeling results confirmed direct correlations among bacterial α-diversity, traits, soil pH, OM, and the C/N ratio (Supplementary Material Fig. S1a). The degree of homogenization of the bacterial community increased from reference forests (79.3 %) to old (83.2 %) and young parks (85.3 %) (Fig. 4a, *x*-axis; Supplementary Material Table S3). This pattern aligns with the variations observed in soil pH, OM, and C/N ratio, consistent with previous findings [83]. This corresponds to a decrease in trait dissimilarity, suggesting homogenization of the bacterial functional trait composition by urban disturbances, thus supporting Hypothesis 3. In contrast, fungal trait composition did not respond clearly to the urban disturbance gradient, although the magnitude of difference in fungal traits was greater in reference forests than in young and old parks (Fig. 4b, *y*-axis). The Δ*β*NTI index suggests that the fungal community is primarily dispersal-limited, indicating that historical contingencies (e.g., ancestral species influencing latecomers and neighboring species [84]) structure this community more than prevailing environmental factors. In other words, the fungal community is primarily influenced by past abiotic or biotic factors rather than the contemporary environment [85], resulting in the lack of clear changes in community and functional composition dispersion (Supplementary Material Table S3). The SEM results also showed that richness (i.e., the number of fungal species), rather than diversity, significantly and positively correlated with fungal traits (i.e., fungal functions correlate with the number of species with little regard to their abundances). This implies that only events that decrease species numbers (e.g., via local extinctions) may reduce the fungal functional potential (Supplementary Material Fig. S1b). Overall, bacterial and fungal communities, with which functional trait composition closely correlates, responded differently to urban disturbances, shaped by historical and abiotic factors. The transition from specialists to generalists in fungi is happening slowly, implying that highly complex forest soils have fewer fungal specialists compared to relatively simple urban soils.

**Fig. 5:**
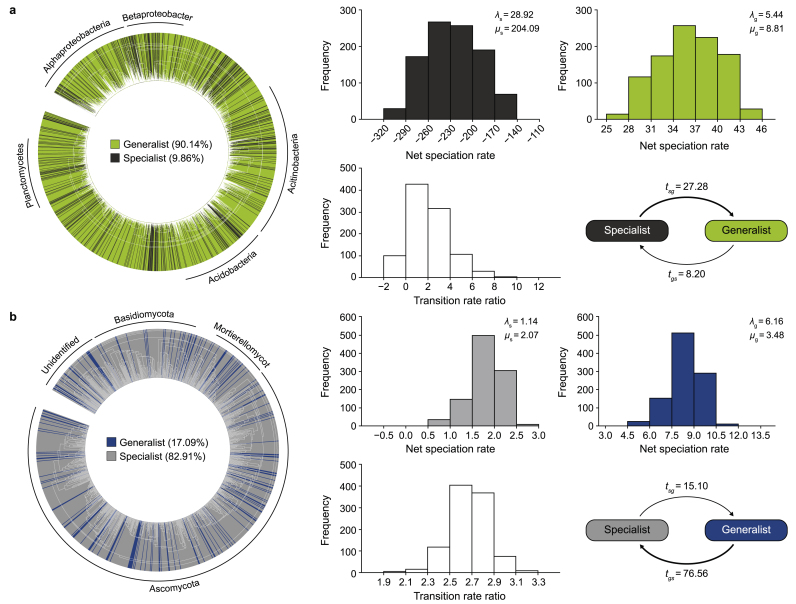
The evolutionary characteristics of bacterial (**a**) and fungal (**b**) communities. Generalists and specialists of bacterial and fungal communities were independent of taxonomic affiliations (phylogenetic trees). The binary-state speciation and extinction (BiSSE) model was used to estimate the evolutionary features of generalists and specialists, including speciation (*λ*), extinction (*μ*), and state-transition (*t*) rates. The net speciation/expansion rate (differences between speciation and extinction rates) of bacterial and fungal communities are showed as histograms (1000-step Markov Chain Monte Carlo simulations) with average speciation and extinction rates. The transition feature is indicated by the ratio (log transformed) of mutual transition rates (*t*_*gs*_/*t*_sg_; *t*_sg_, from specialist to generalist, *t*_gs_, from generalist to specialist) and average transition rates of bacterial and fungal communities.

The divergent structuring of bacterial and fungal communities, as well as their functional potentials, may be influenced by evolutionary forces, such as speciation and local extinction. We used the BiSSE model [76] to classify taxa into generalists (adaptive to diverse environments) or specialists (thrive in specific environments), such that generalists can exist in a greater diversity of environments than specialists (Fig. 5, left panels). Our analyses categorized 90.14 % of bacteria as generalists, indicating a substantial bacterial resilience to diverse environments due to a significantly higher transition rate from specialists to generalists (*t*_sg_ = 27.28) compared to the reverse (*t*_gs_ = 8.20) (Fig. 5a), suggesting a high bacterial resilience to diverse environments. The higher extinction than speciation rate of generalist bacteria in urban parks compared to reference forests suggested that urban disturbances compositionally restructure the bacterial communities by reducing the number of generalists. This trend was supported by the declining transition rates (*t*_sg_/*t*_gs_, transition from specialist to generalist vs. the reverse) along the urban disturbance gradient from reference forests (25.69) to old (0.92) and to young parks (0.013) (Supplementary Material Table S4). This indicates that a wide range of functions that flourish in reference forests decline in urban systems.

In our analysis of fungal communities across urban parks and reference forests, we observed a dominant specialist ratio (82.91 %) and a more pronounced generalist-to-specialist shift (*t*_gs_ = 76.56) compared to the reverse (*t*_sg_ = 15.10) (Fig. 5b). While this indicates greater fungal specialization to local/regional environments compared to bacteria, it does not necessarily equate to higher vulnerability. Factors other than specialization might contribute to the observed fungal stability in the face of urban disturbances. The increasing net specialist speciation rate (*λ*_s_−*μ*_s_) from reference forests (3.62) to old (3.85) and young parks (11.59) (Supplementary Material Table S4) suggests that fungal communities appear to have more specialists than generalists in urban environments, suggesting that fungal species that have ecological roles specific to urban conditions accumulate due to urban disturbances. This indicates that the composition of bacterial and fungal communities reacts differently to urban disturbances, with urban habitats favoring fungal specialists and bacterial generalists.

The differential responses of bacterial and fungal communities in urban versus forested environments could be influenced by the nature of available substrates [86]. Reference forests often harbor complex organic materials requiring specialized enzymatic processes for decomposition, which might favor fungal specialists capable of breaking down these substrates [87]. Conversely, urban environments, especially younger ones, might have simpler, more readily available carbon sources stemming from anthropogenic inputs, favoring bacterial generalists that can rapidly exploit these sources [88]. This juxtaposition of substrate complexity and availability could underpin the observed trend of increasing fungal specialization in urban environments [89]. Further studies dissecting the exact nature of organic matter and carbon sources across these habitats would shed more light on this hypothesis.

### 3.3 Functional potentials

Ecologists often assume that microorganisms exhibit uniform adaptive strategies across diverse environments due to their small size, short generation times, and high dispersal potential [84,90]. However, our study challenges this notion, particularly when distinguishing between bacterial and fungal responses to urban disturbances. While both communities exhibited increased homogenization from reference forests to old and young urban parks (Hypothesis 1), their responses differed significantly (Hypothesis 2). Notably, the variation in bacterial and fungal functional traits exceeded that of their taxonomic composition (Hypotheses 3 and 4). In bacterial communities, the disturbance gradient, influenced by factors such as soil pH and urban management practices [91], may drive local extinctions [92]. Managed systems, such as turf grass, often experience an increase in soil carbon, while unmanaged disturbances typically reduce it. Urban habitat characteristics, particularly management strategies, are pivotal in shaping microbial and fungal environments. Consequently, while functional traits of certain bacterial taxa may be lost, many redundant traits persist in the urban ecosystem. This loss of functional potential likely explains the greater compositional homogenization observed in urban bacterial communities compared to their functional potential, which we attribute to redundancy—the diversity that maintains functional potential.

In contrast to bacterial communities, the structuring of fungal communities appears to be more influenced by the concept of “ecological memory”—the influence of past events on the present community state [93]. According to Sydenham et al. [94], fungal communities exhibit limited dispersal abilities and undergo slower transitions from specialist to generalist roles than bacterial communities. This resilience is evident in urban parks, where fungal communities maintain composition despite urbanization pressures [95,96]. However, the term “urbanization” encompasses direct disturbances, management practices, and broader environmental impacts, including rising temperatures and nitrogen deposition. Even in urban parks, where fungal specialists decline, nearby forests are not immune to urban influences. The emergence of adaptable generalists, potentially driven by fungi's extensive genetic diversity, suggests a gradual shift in fungal communities, underscoring their sensitivity to urban-related changes.

In this study, the community structures of bacteria and fungi differed in their responses to urban disturbances. Bacteria, with their small propagule size, dispersed more easily and appear more sensitive to abiotic factors than fungi [97]. Additionally, urban soils contained at least 10 times more abundant and species-rich bacterial populations than fungi, potentially facilitating functional redundancy. Furthermore, despite changes in the composition of these microbial communities due to urbanization, their overall functions remained consistent. Urban disturbances had a more significant impact on fungal dispersal than bacteria dispersal. The decay rate of community similarity with geographic distance has been thoroughly researched across ecosystems [98,99]—with fungi, particularly, exhibiting strong spatial structuring [78,100]. Our findings indicate that urban disturbances can affect fungal community composition even at small spatial scales, an observation likely attributable to dispersal limitation.

## 4 Conclusions

Human-driven disruption to natural environments, particularly their conversion to urban greenspaces, can reduce soil microbiome diversity and alter its composition and functional potential. Ecosystem functions rely on the organisms present in the system. Therefore, when the habitat is disrupted, the survival of these functions depends on the resistance and resilience of the resident organisms and their specific survival strategies. Microbial communities possess exceptional diversity and redundant metabolic versatility, which help sustain essential ecosystem functions, such as carbon and nutrient cycling. Based on our data, urban disturbances filter out taxa with different functional attributes or enhance the environmental tolerance of particular ones, leading to an asynchronous homogenization of microbial composition and functional traits. This process can aid in maintaining life-supporting functions in highly disturbed urban soils.

## CRediT authorship contribution statement

**Bangxiao Zheng:** Writing – review & editing, Writing – original draft, Visualization, Validation, Software, Methodology, Investigation, Formal analysis, Data curation, Conceptualization. **Nan Hui:** Writing – review & editing, Writing – original draft, Project administration, Investigation, Funding acquisition, Conceptualization. **Ari Jumpponen:** Writing – review & editing, Validation, Supervision. **Changyi Lu:** Writing – review & editing, Methodology, Investigation. **Richard Pouyat:** Writing – review & editing. **Katalin Szlavecz:** Writing – review & editing. **David A. Wardle:** Writing – review & editing. **Ian Yesilonis:** Writing – review & editing, Methodology, Investigation. **Heikki Setälä:** Writing – review & editing, Methodology, Investigation. **D. Johan Kotze:** Writing – review & editing, Investigation.

## Declaration of competing interest

The authors declare that they have no known competing financial interests or personal relationships that could have appeared to influence the work reported in this paper.
