# Supplementary material for: Urbanization leads to asynchronous homogenization of soil microbial communities across biomes
Source: Environ Sci Ecotechnol. 2025 Mar 17;25:100547. doi: 10.1016/j.ese.2025.100547 (PMC11987689; doi:10.1016/j.ese.2025.100547)
Supplement: Multimedia component 1 [file mmc1.pdf]

Supplementary Materials for

Urbanization leads to asynchronous homogenization of soil microbial communities across biomes

Bangxiao Zheng *et al.*

*Corresponding author. Email: nan.hui@sjtu.edu.cn

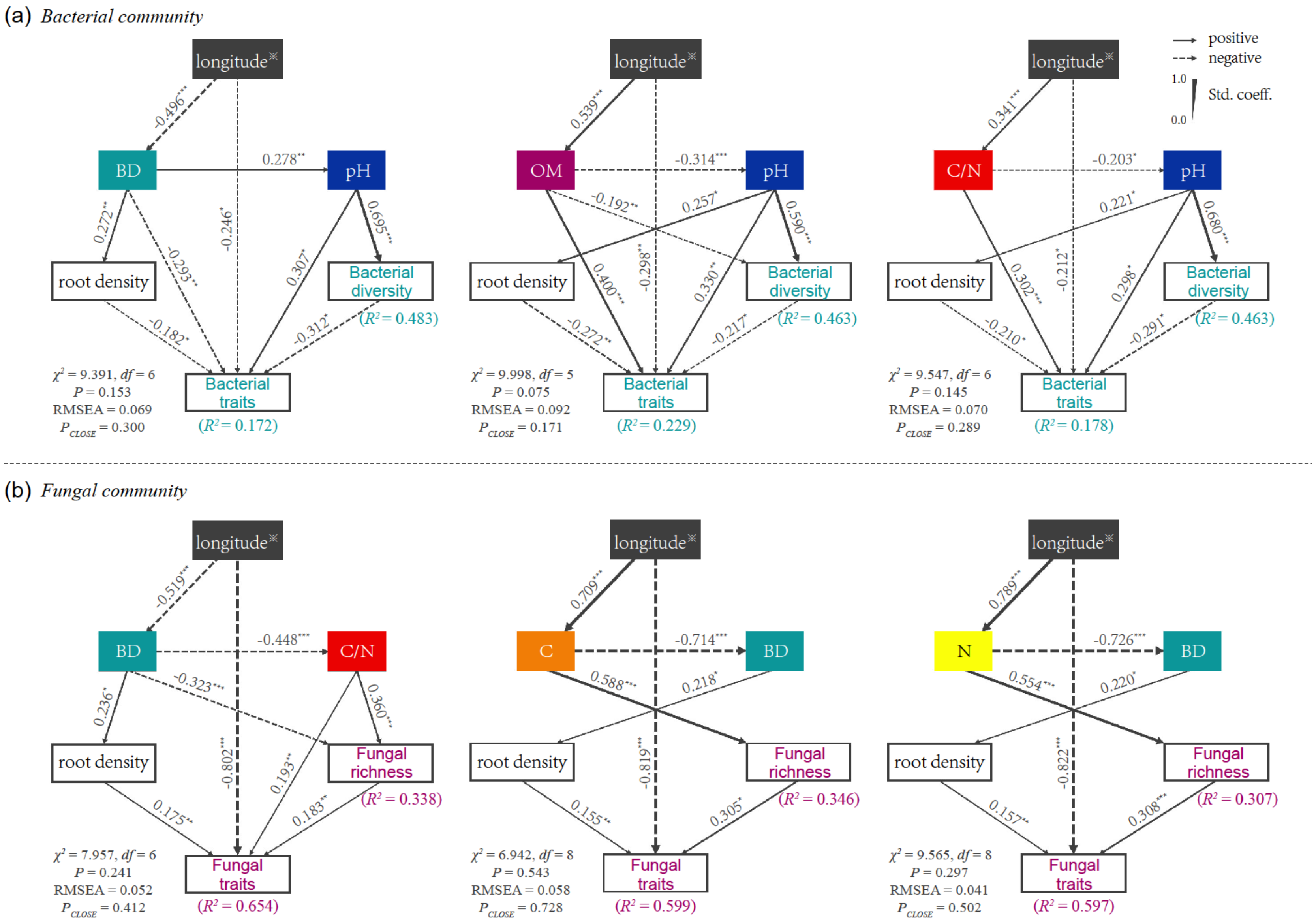

Fig. S1. SEMs analyzing the relationships between edaphic properties, root density, taxonomic diversity, and trait abundance of bacterial (a) or fungal (b) communities. Std. coeff., standard coefficient; BD, bulk density; OM, organic matter; C/N: carbon to nitrogen ratio; ※Longitude was transformed to distance to the equator. Different combinations of these variables were tested, but only three combinations in bacterial and fungal communities satisfied goodness of fit standards.

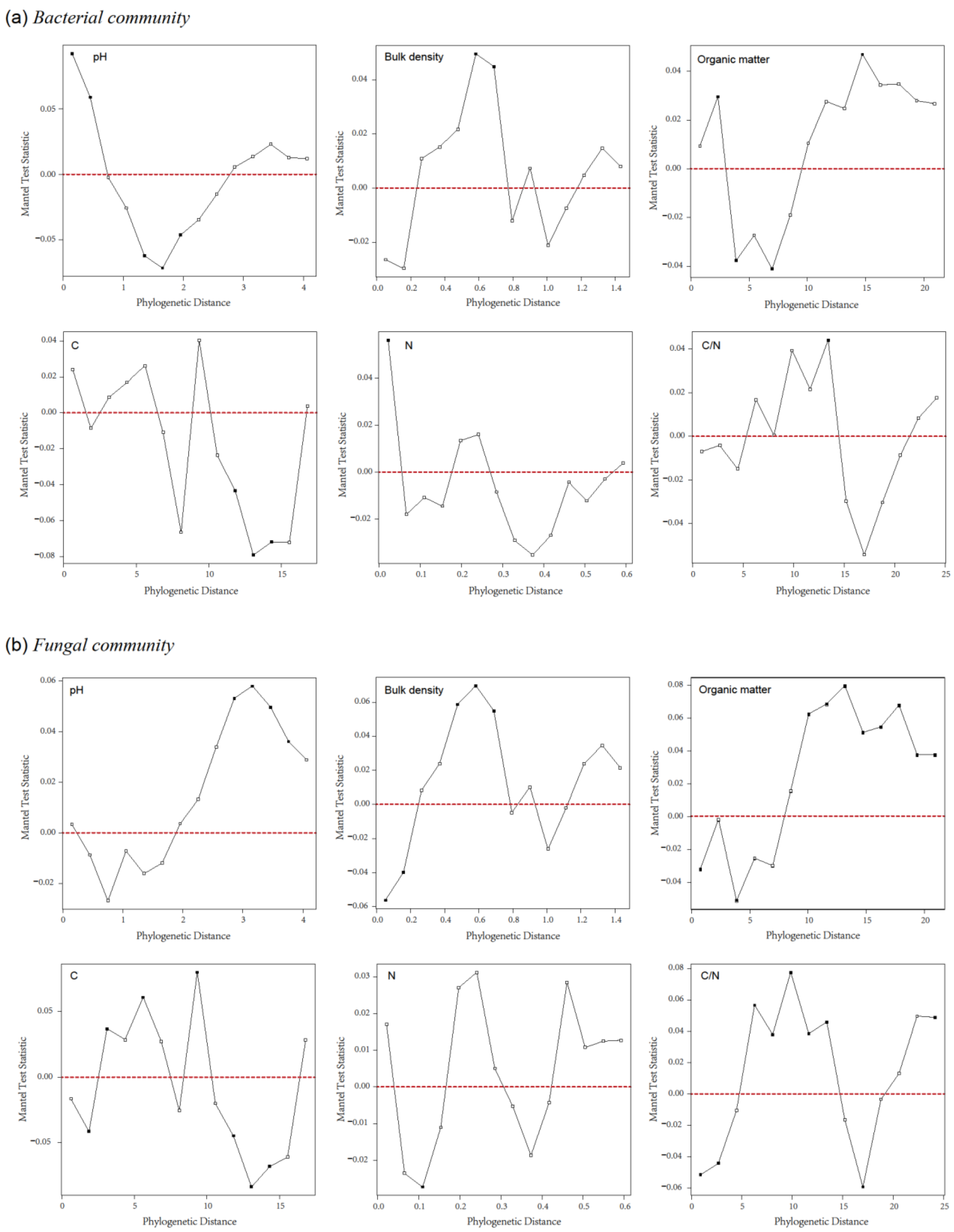

Fig. S2. Phylogenetic Mantel correlogram showing phylogenetic signals of the bacterial and fungal communities across phylogenetic distances. Closed and open dots denote significant and non-significant correlations, respectively, relating between-OTU niche differences to between-OTU phylogenetic distances across a given phylogenetic distance. Estimates of optimal OTU environmental niches were calculated for soil pH, bulk density, organic matter, C content, N content, and the C/N ratio. Significantly positive correlations indicate that ecological niche distance between OTUs increases with phylogenetic distance, but only across the phylogenetic distance class evaluated (i.e., there is a phylogenetic signal in OTU environmental niches). All edaphic properties indicate significant correlations at different phylogenetic distances.

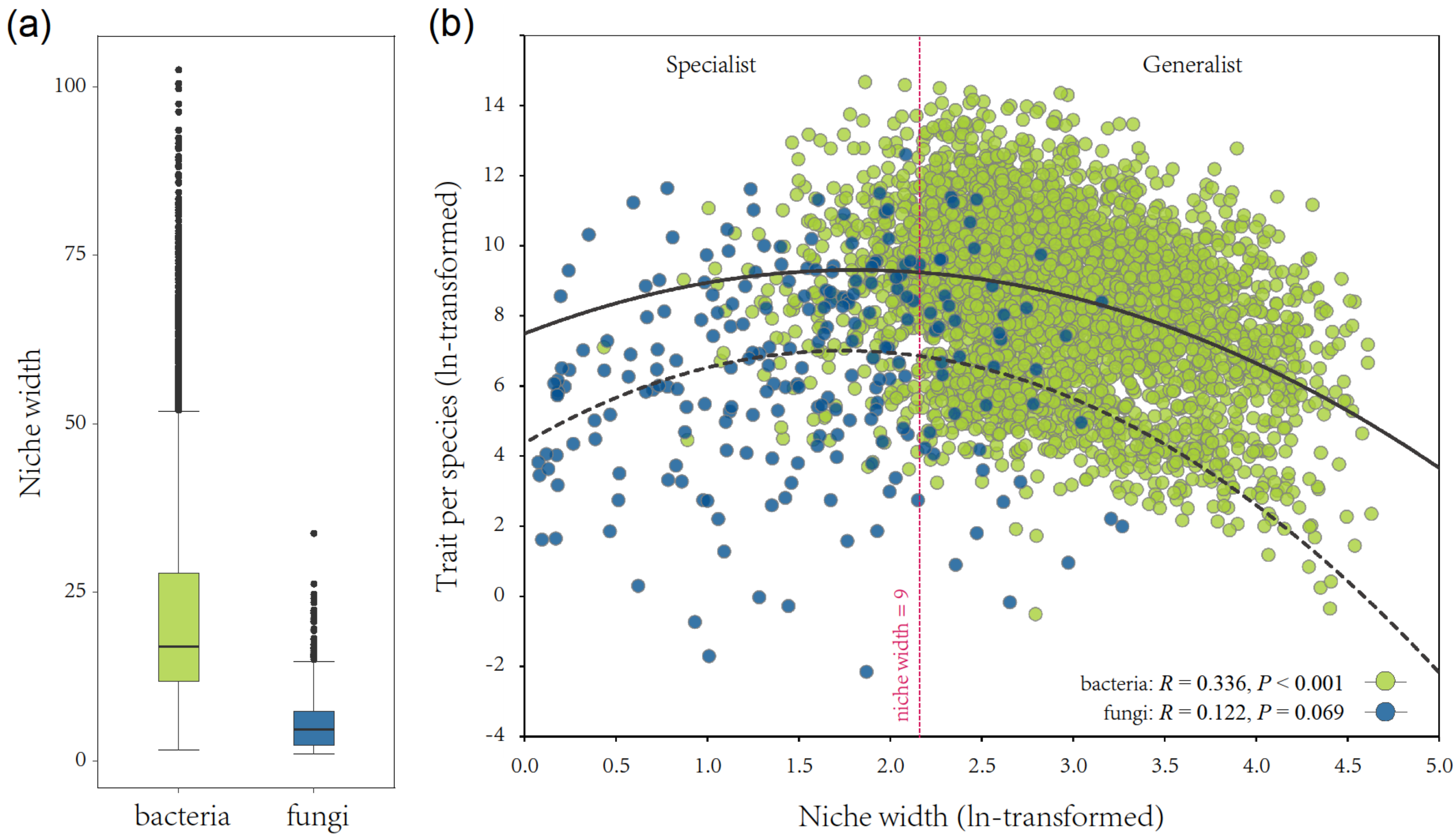

Fig. S3. Niche widths of bacterial and fungal communities (a) and their relationships with trait per species (b). Niche width 9 was chosen as a demarcation point (Methods)

Table S1. Effects of biome, urbanization, and vegetation type on microbial taxa and trait composition using PERMANOVA. Each factor — biome, urbanization, and vegetation type — was analyzed independently by controlling for the other variables. For example, in assessing the effect of biome, statistical models were adjusted to account for all categories of urbanization and vegetation types, thus isolating the biome effect.

		**Biome**			**Urbanization**			**Vegetation type**		
		F value	R^2	*P*	*F* value	R^2	*P*	*F* value	R^2	*P*
	Taxon composition									
Bacteria		26.075	0.308	0.001	2.813	0.046	0.002	1.083	0.018	0.299
Fungi		11.327	0.162	0.001	2.570	0.042	0.001	1.281	0.021	0.036
	Trait composition									
Bacteria		11.006	0.158	0.001	3.729	0.060	0.006	0.786	0.013	0.590
Fungi		24.130	0.292	0.001	1.683	0.028	0.059	0.786	0.013	0.655
Total		16.807	0.223	0.001	3.759	0.060	0.001	0.688	0.012	0.728

Table S2. The effects of biome and urbanization on microbial taxon and trait composition by PERMDISP.

	**Effect of biome**					**Effect of urbanization**				
	F	*P (perm)*	TP *vs.* BR	TR *vs.* BR	TR *vs.* TP	*F*	*P (perm)*	R *vs.* O	Y *vs.* O	Y *vs.* R
Taxon composition										
Bacteria	10.092	0.001	0.834	<0.001	0.001	6.874	0.003	0.016	(0.636)	(0.001)
Fungi	1.284	0.257	(0.821)	0.586	0.255	1.948	0.144	(0.818)	(0.129)	(0.491)
Trait composition										
Bacteria	6.567	0.003	(0.013)	(0.003)	(0.890)	17.627	0.001	<0.001	(0.276)	(<0.001)
Fungi	17.247	0.001	(0.648)	<0.001	<0.001	1.306	0.265	0.419	(0.939)	(0.265)
Total	4.361	0.013	(0.017)	(0.073)	0.827	18.296	0.001	<0.001	(0.307)	(<0.001)

Note: The value of each comparison is the contrast *P* value by Tukey multiple comparisons of means. With or without parenthesis indicates that the difference of variability is negative or positive (from the left to right side of "*vs.*"). *P* (*perm*) values are permutation-based *P* values for the overall PERMDISP analysis of each outcome. Abbreviations of sites: TP, temperate climate; BR, boreal climate; TR, tropical climate; R, reference forest; O, old park; Y, young park.

Table S3. Summary of the relative importance (%) of different ecological processes.

Process	Criterion		Bacterial community			Fungal community		
	bNTI	RC_{bray}	R	O	Y	R	O	Y
Determinism								
Homogeneous selection	<-2	-	79.31	83.23	85.25	8.28	5.86	11.21
Heterogeneous selection	>2	-	0.46	1.21	4.04	3.22	0.61	1.21
Stochasticity								
Dispersal limitation	[-2, 2]	>0.95	17.01	10.30	8.08	78.85	84.85	72.02
Drift	[-2, 2]	[-0.95, 0.95]	1.15	3.43	1.11	9.20	8.59	15.15
Homogenising dispersal	[-2, 2]	<-0.95	2.07	1.82	1.52	0.46	0.10	0.40

Note: *Determinism* and *Stochasticity* refer to deterministic and stochastic processes of evolution driving the structure of microbial communities by the abiotic environment or by chance colonization and random extinction, respectively (Chase & Myers, 2011). The pairwise comparison of 120 samples generated 7 140 bNTI and RC_{bray} values each. bNTI: β-nearest taxon index; RC_{bray}: Raup-Crick metric based on species Bray-Curtis dissimilarity. R = reference forests, O = old parks, Y = young parks.

Table S4. Estimation of evolutionary characteristics of bacterial and fungal communities with urbanization.

	Reference forests	Old parks	Young parks
Bacterial community			
Net speciation rate of specialists (λ_s-μ_s)	-0.36	0.25	0.25
Net speciation rate of generalists (λ_g-μ_g)	0.75	0.57	0.58
Ratio of state transition rate (t_{sg}/t_{gs})	25.69	0.92	0.013
Fungal community			
Net speciation rate of specialists (λ_s-μ_s)	3.62	3.85	11.59
Net speciation rate of generalists (λ_g-μ_g)	1.48	1.27	-0.14
Ratio of state transition rate (t_{sg}/t_{gs})	0.15	0.17	0.18

Note: Values shown are averages. Stability and robustness of the estimated rates were assessed with 1 000 steps Markov Chain Monte Carlo simulations. λ and μ are speciation and extinction rates, whereas *g* and *s* represent generalist and specialist states, respectively. *t* indicates the state transition rate.
